# Supplementary material for: Computer-Aided Detection of Quantitative Signatures for Breast Fibroepithelial Tumors Using Label-Free Multi-Photon Imaging
Source: Molecules. 2022 May 23;27(10):3340. doi: 10.3390/molecules27103340 (PMC9144626; doi:10.3390/molecules27103340)
Supplement: Supplementary file 1 [file molecules-27-03340-s001.zip › molecules-1707628-SI.pdf]

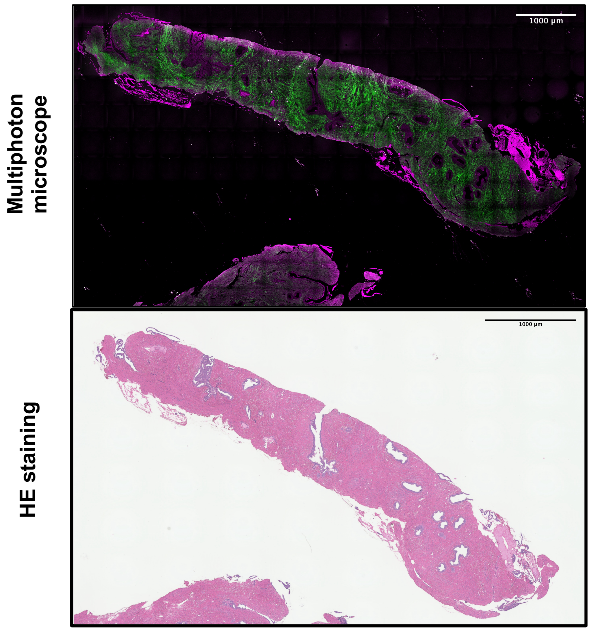

Supplementary Figure S1. Whole view images of needle biopsied tissue sections with multi-photon microscopy and HE stained samples. Several fields indicated as white frames were 1 mm × 1 mm FoV images, and were chosen witout overlap and almost the whole region was filled when combining those together. Scale bar, 1 mm.

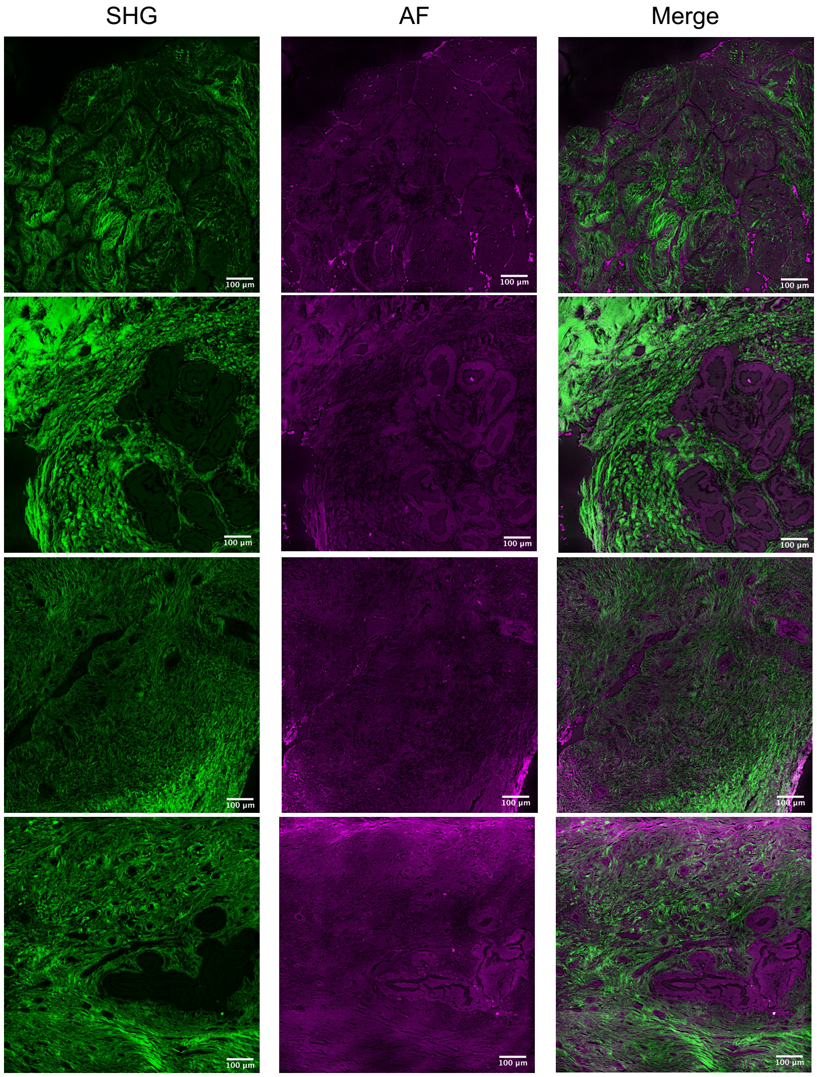

Supplementary Figure S2. Multi-photon microscopy images of tissue sections for FA lesions. Each image was from different patient samples. Scale bar, 100 µm.

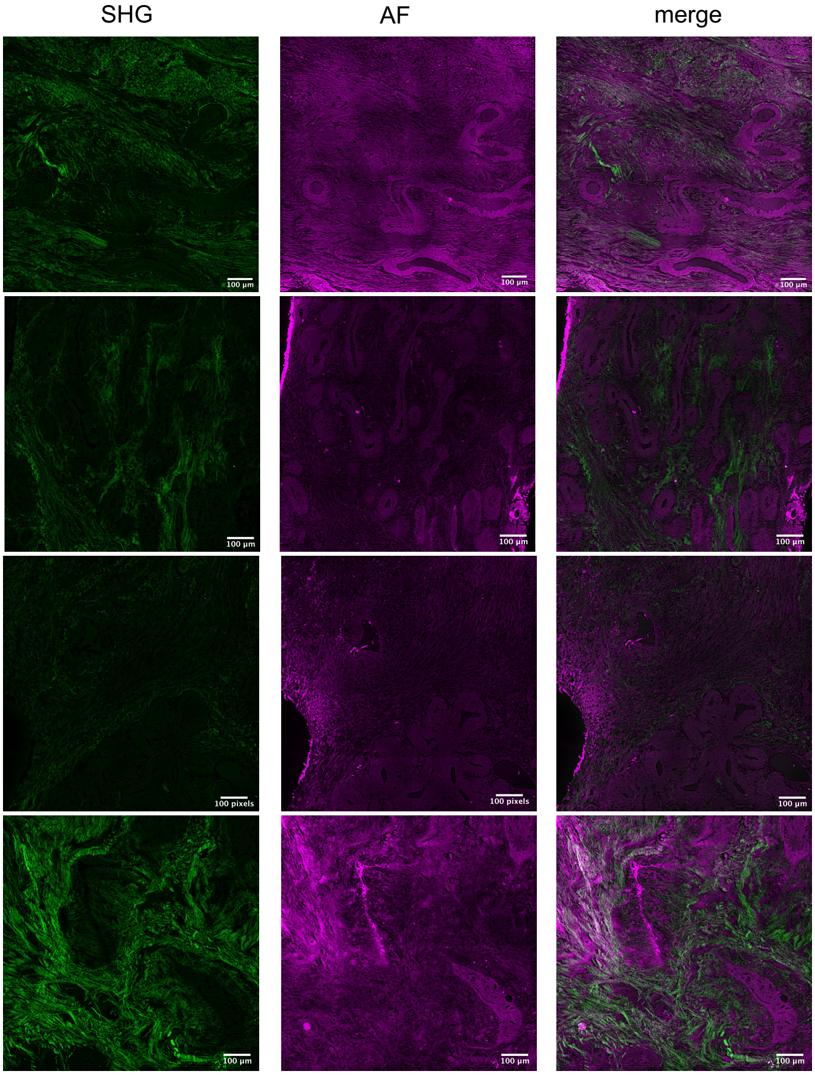

Supplementary Figure S3. Multi-photon microscopy images of tissue sections for PT lesions. Each image was from different patients samples. Scale bar, 100 µm.

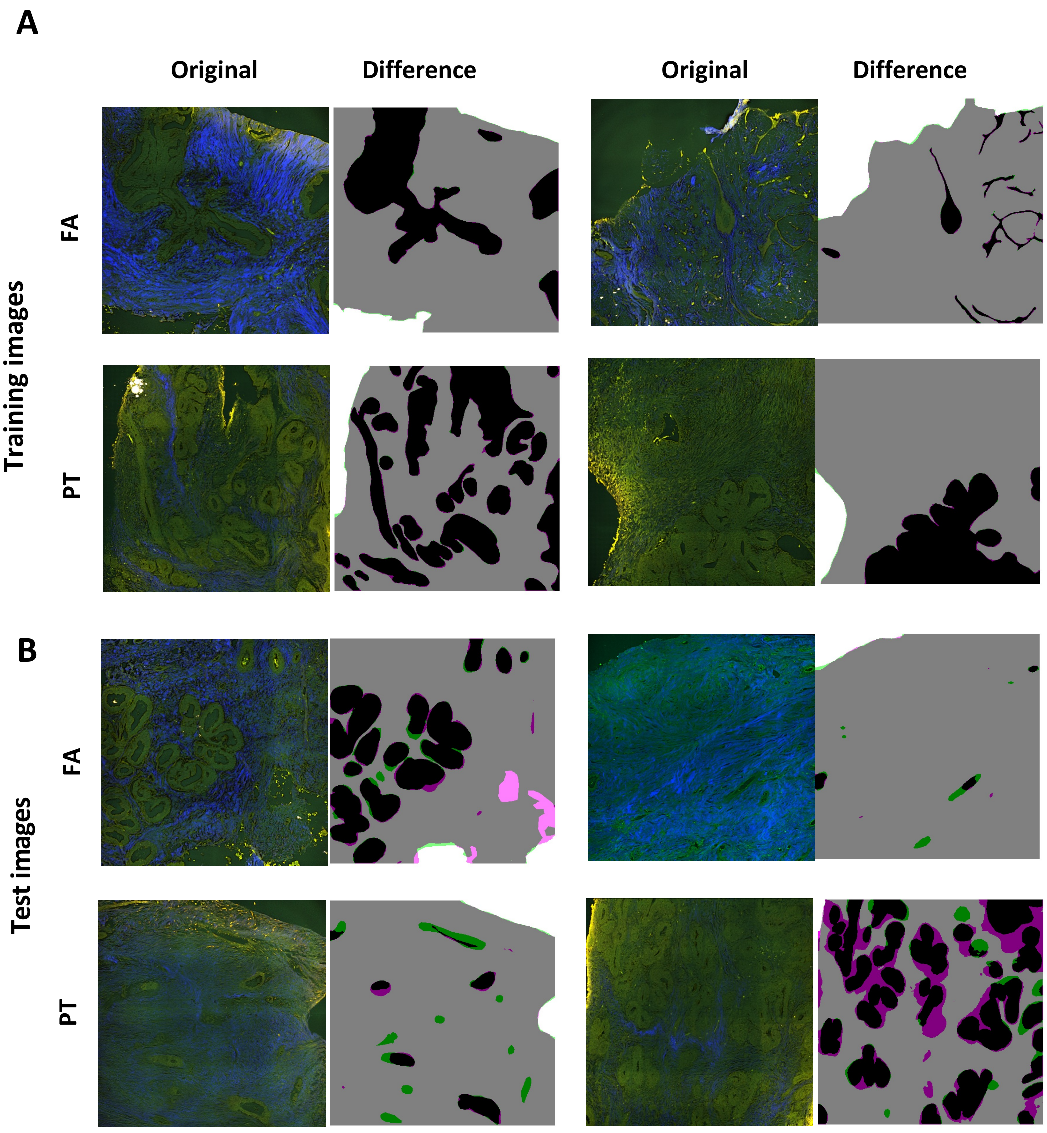

Supplementary Figure 4. Results of image segmentation by a deep learning based framework, SegNet. (A) Results of training image sets. Original multi-photon microscopy images and difference images between ground-truth and predicted images were shown for both FA and PT images. Differences in images indicated FN areas as magenta and FP areas as green. (B) Results of test image sets.

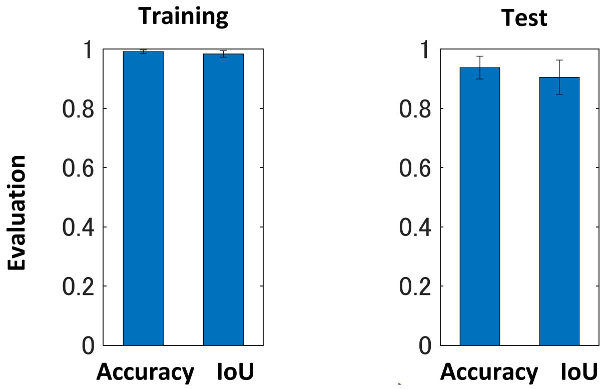

Supplementary Figure S5. Numerical evaluation of the segmentation results for the case where another 50% randomly selected images were used for training. The total accuracy between the ground-truth and predicted images, and the weighted IoU are shown for training and test data sets. These scores are evaluated for each image in training and test cases, and statistics such as mean and standard deviation are calculated. The bar denotes average; the error bar denotes standard deviation over the data calculated from image sets.

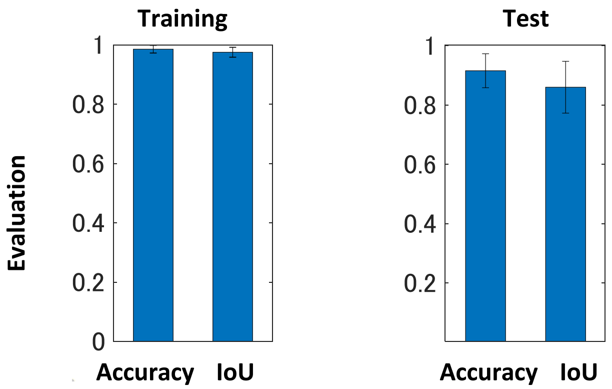

Supplementary Figure S6. Numerical evaluation of the segmentation results for the case where 20% randomly selected images were used for training. The total accuracy between the ground-truth and predicted images, and the weighted IoU are shown for training and test data sets. These scores are evaluated for each image in training and test cases, and statistics such as mean and standard deviation are calculated. The bar denotes average; the error bar denotes standard deviation over the data calculated from image sets.

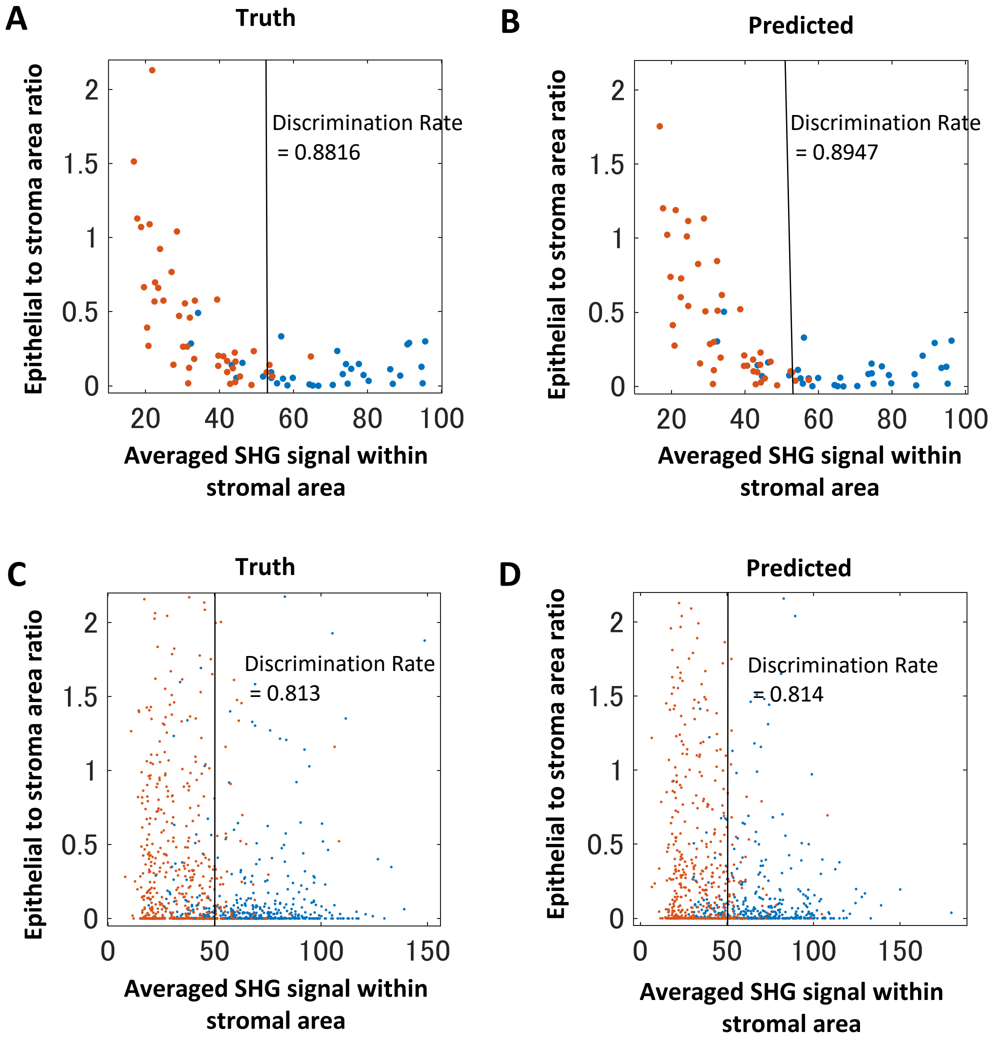

Supplementary Figure S7. Scatter plots and discrimination analysis of the two quantification scores. (A,B) Scatter plot of the ground-truth (A) and the predicted (B) data for original (512 ×512 px) image set. Blue and red circles denote FA and PT data, respectively. The lines represent the fitted lines for discrimination between FA and PT scores. (C,D) Scatter plot of the ground-truth (A) and the predicted (B) data for small size (128 x 128 px) image set. The small size image sets are generated by dividing the original (512 x 512 px) image into 4x4 blocks. From these images, 500 images are randomly selected (excluding the stromal area = 0 block) for both FA and PT. Score calculation is performed for each image. Blue and red circles denote FA and PT data, respectively. The lines represent the fitted lines for discrimination between FA and PT scores.
